# Qualitative testing of the EQ-HWB-9 with a ‘blank’ EQ-HWB VAS and EQ-5D-5L with skin bolt-ons in a rare genetic skin disease population

**DOI:** 10.1007/s11136-026-04177-0

**Published:** 2026-02-12

**Authors:** Zseraldin Metyovinyi, Dóra Plázár, Krisztina Becker, Márta Medvecz, Fanni Rencz

**Affiliations:** 1 Department of Dermatology, Venerology and Dermatooncology, Semmelweis University, 41 Mária Street, Budapest, 1085 Hungary; 2 Department of Health Policy, Corvinus University of Budapest, Budapest, Hungary; 3 EuroQol Research Foundation, Rotterdam, The Netherlands

**Keywords:** Ichthyosis, Epidermal differentiation disorder, EQ-5D-5L, EQ-HWB-9, Bolt-on

## Abstract

**Purpose:**

Epidermal differentiation disorders (EDDs) are rare genetic skin disorders that significantly impact patients’ health and wellbeing. This study assessed the content validity of two experimental EuroQol instruments (EQ-5D-5Lbolt-ons and EQ-HWB-9) and explored potential endpoint options for a visual analogue scale (VAS) for EQ-HWB-9 in this population.

**Methods:**

Semi-structured interviews were conducted with 20 adult patients with EDDs at a university dermatology clinic (2024–2025). Participants completed the EQ-5D-5L with skin irritation and self-confidence bolt-ons and EQ-HWB-9 using a think-aloud protocol. Probing questions explored item relevance, missing concepts, and comprehensibility. An EQ-HWB VAS with endpoints blanked out was also presented for feedback.

**Results:**

Participants found both instruments generally comprehensive, understandable, and relevant for EDD. The EQ-5D-5L with bolt-ons was considered suitable for measuring health- and disease-related aspects (e.g., skin irritation bolt-on was the most relevant item for all but one patients), while the EQ-HWB-9 was seen as relevant to measure broader life impacts. Three participants suggested combining the EQ-HWB-9 loneliness item with others, and three participants recommended splitting the sadness/depression item. One participant noted missing social/romantic relationship items in both instruments. Overall, 32 different concepts were proposed as EQ-HWB VAS endpoint labels, including both health-related and broader wellbeing concepts, such as overall feeling, mood, quality of life, and harmony.

**Conclusion:**

Both the EQ-5D-5L with bolt-ons and EQ-HWB-9 demonstrated acceptable content validity in this genetic disease population. Our findings provide useful input for finalizing these experimental instruments and offer the first exploratory qualitative results about potential endpoints for an EQ-HWB VAS.

**Supplementary Information:**

The online version contains supplementary material available at 10.1007/s11136-026-04177-0.

## Introduction

Health-related quality of life (HRQoL) is an important outcome in daily clinical practice, clinical trials, observational studies, as well as in economic evaluations. The EQ-5D-5L is the most widely used generic, preference-accompanied measure of HRQoL [1–3]. Although it has shown acceptable reliability and validity in various dermatological diseases, its sensitivity may be limited. As a generic instrument with five broad dimensions (mobility, self-care, usual activities, pain/discomfort, and anxiety/depression), it may not capture all important impacts of skin diseases, such as skin irritation and itching, and therefore it may not fully reflect all treatment-related changes on HRQoL [4–11]. Two skin-specific bolt-ons (skin irritation and self-confidence) have been developed to enhance the EQ-5D-5L’s ability to measure key aspects of HRQoL in individuals with psoriasis [12–15]. The two bolt-ons are currently included in the experimental EQ-5D Bolt-on Toolbox™. Recent studies have found that these bolt-ons are valid and improve the measurement properties of the EQ-5D-5L in other skin conditions, such as atopic dermatitis, chronic urticaria, Darier’s disease, Hailey-Hailey disease, and minimally invasive cosmetic skin procedures [16–21].

To cover broader aspects beyond the traditional boundaries of HRQoL, the EuroQol Health and Wellbeing (EQ-HWB) measure has recently been developed [22]. The EQ-HWB aims to assess a broader range of outcomes, encompassing both health-related items (e.g., day-to-day activities, pain, anxiety) and broader aspects, such as control and loneliness, which are particularly important in the context of caregiving and social care. As a common outcome measure suitable across healthcare and social care sectors, the EQ-HWB is intended to facilitate cross-sector decision-making. Two versions of the instrument are available: the long form, EQ-HWB, and the short form, EQ-HWB-9. Similarly to the two skin bolt-ons, both the EQ-HWB and EQ-HWB-9 are currently experimental instruments under development. So far, given the scope of the measure, most validation studies have been conducted in caregivers, social care users, and general population samples [23–33]. Only a few studies have documented their measurement properties in targeted patient populations, primarily focusing on cancer [34–38].

Epidermal differentiation disorders (EDDs), previously known in clinical practice as ichthyoses, are rare skin conditions that are genetically and clinically diverse [39–41]. EDDs are classified into non-syndromic forms (nEDD), limited to the skin and its appendages, and syndromic forms (sEDD), which often involve extracutaneous manifestations [42–46]. Treatment is generally symptomatic, focusing on barrier repair and symptom relief using emollients, keratolytics, retinoids, or other repurposed agents [47, 48].

EDDs substantially impact patients’ HRQoL, quality of life, and wellbeing. These impacts include skin symptoms, such as itching and pain, as well as various psychological problems, such as low self-esteem, anxiety, and depression. Social impacts may include stigmatization, discrimination, and difficulties at the workplace, in relationships, and financial wellbeing [49–56]. Both EQ-5D-5L with bolt-ons and EQ-HWB-9 may be useful in EDDs; however, they serve different purposes, given the distinct aims of each. The bolt-ons may be relevant in EDDs due to the skin symptoms and related potential self-confidence issues, while the EQ-HWB-9, by measuring broader aspects than HRQoL, seems relevant in this lifelong genetic disorder to capture the overall life impact.

This study, therefore, aimed to investigate the content validity (relevance, comprehensiveness, and comprehensibility) of the EQ-5D-5L with two bolt-ons (skin irritation and self-confidence) from the EQ-5D Bolt-on Toolbox and the EQ-HWB-9 among patients with EDDs. A secondary objective was to explore adding a visual analogue scale (VAS) to the EQ-HWB-9 and to identify possible endpoint labels for the scale.

## Methods

### Participants, study design

The study design and data analysis followed the Standards for Reporting Qualitative Research checklist [57]. A regional institutional research ethics committee approved the research (No. 220-1/2022, SE RKEB). Qualitative, semi-structured one-on-one interviews were conducted with Hungarian patients diagnosed with EDD (nEDD or sEDD). All EDD subtypes were considered and analyzed together due to their similar clinical manifestations and treatment regimens, which result in comparable HRQoL impairment. Between December 2024 and May 2025, patients were enrolled at a university dermatology clinic in Budapest, Hungary. Inclusion criteria for the study were (1) age ≥ 18 years; (2) clinical or genetic diagnosis of nEDD or sEDD; (3) absence of cognitive impairments; (4) ability to understand the questions in Hungarian; and (5) written informed consent. The study included participants until data saturation was reached (i.e., when no new important themes emerged in the last three interviews) [58]. Disease severity was assessed using validated objective severity scales, including the Ichthyosis Area Severity Index (IASI) and the Visual Ichthyosis Severity Index (VISI) [59, 60].

### EQ-5D-5L and bolt-ons

The EQ-5D-5L consists of two parts: a five-dimensional descriptive system and a visual analogue scale, the EQ VAS [61]. In our study, two previously developed bolt-ons, skin irritation and self-confidence, were administered before the EQ VAS [12]. While all existing EQ-5D-5L bolt-ons were considered, we selected skin irritation and self-confidence from the experimental EQ-5D Bolt-on Toolbox (EV1.0) for this study due to their established psychometric performance in other dermatological conditions and their potential relevance to EDD based on the literature [15–21, 50]. Each dimension of the descriptive system and bolt-ons uses a single-item with a severity scale ranging from ’no problems’ to ’unable to’/’extreme problems’. The EQ VAS is a vertical scale ranging from 0 (representing ‘the worst health you can imagine’) to 100 (representing ‘the best health you can imagine’). The day of completion (’today’) is the recall period for the descriptive system, bolt-ons, and EQ VAS. The official Hungarian self-complete versions were used for both the EQ-5D-5L and the bolt-ons.

### EQ-HWB-9 and EQ-HWB VAS

The EQ-HWB-9 consists of a 9-item descriptive system [22]. In this study, the Hungarian self-complete ‘experimental modified’ (2024) version was used. The nine items, in the order of appearance, include day-to-day activities, getting around inside or outside, exhaustion, loneliness, concentrating/thinking clearly, anxiety, sadness/depression, control, and physical pain. The first two items use a five-point difficulty scale ranging from ‘no difficulty’ to ‘unable’. Items 3 to 8 use a frequency scale ranging from ‘none of the time’ to ‘most or all of the time’, and the pain item uses a severity scale ranging from ‘no physical pain’ to ‘very severe physical pain’. The timeframe for each item is the last seven days.

While the EQ-5D-5L includes a VAS as part of the instrument (EQ VAS), this has never been proposed for the EQ-HWB or EQ-HWB-9. Including a VAS after the EQ-HWB-9 could provide a single metric capturing a respondent’s overall perspective, incorporating both health and broader aspects. Its use could provide a person-centered numeric score applicable across the contexts for which the instrument is designed, capture information that may not be fully reflected in the descriptive system, and enhance the instrument’s value, including its potential use in future psychometric validation. Yet, it remains unclear how such an EQ-HWB VAS would look like, including the appropriate endpoint labels. To explore this further, we developed a ‘blank’ EQ-HWB VAS with a 7-day recall period, matching the EQ-HWB-9, and in terms of layout and format, completely resembling the EQ VAS. However, we removed any references to health and instead used a blank space (dotted line) to facilitate discussions with patients about what a VAS for the EQ-HWB-9 should measure and what the potential endpoints might be.

### Interviews

All interviews were conducted by the first author. They were either face-to-face in a quiet private room at the dermatology clinic or online via video calls, depending on the participant’s preference. A topic guide was developed by the research team, building on three similar studies in other dermatological populations [16, 17, 19].

At the beginning of the interview, the research aims and interview process were clearly explained to the participants. The interviews then focused on briefly exploring the key aspects of health, quality of life, and wellbeing affected by their condition (hereafter we refer to this section as ’concept elicitation’). Participants subsequently completed the EQ-5D-5L descriptive system with two bolt-ons, followed by the EQ VAS, and the EQ-HWB-9, while ’thinking aloud’ to explain their reasoning as they responded [60]. After completing the EQ-HWB-9, they were presented with the ’blank’ EQ-HWB VAS and were asked how they would complete the dotted lines to align them with the EQ-HWB-9. Importantly, the participants did not receive any prior explanation about the purposes of the two instruments.

After finishing each questionnaire, in line with the Consensus-based Standards for the selection of health Measurement Instruments (COSMIN) guidelines, exploratory questions were used to evaluate three aspects of content validity: relevance, comprehensiveness, and comprehensibility [63, 64]. Participants were asked to identify any overlaps or missing concepts and assess the clarity of item wording, response options, and appropriateness of the recall period. The response levels changed from the ‘experimental’ (2022) to the ‘experimental modified’ (2024) version of the EQ-HWB-9 were probed to explore these differences (‘only occasionally’ vs. ‘a little of the time’ and ‘some difficulty’ vs. ‘moderate difficulty’). Furthermore, participants were asked to compare the EQ-5D-5L with two bolt-ons and the EQ-HWB-9 using their own words, along with targeted questions on content, recall period (e.g., today vs. last week), response scales (i.e., severity vs. difficulty/frequency), format (e.g., item-by-item vs. grid), and suitability in capturing the impacts of disease on their lives. In the last section of the interview, patients completed a short questionnaire about their sociodemographic and clinical background (sex assigned at birth, age, education, age at onset of first symptoms, comorbidities, number of affected body areas, and current treatments).

### Data analysis

Every interview was completed anonymously, and audio recordings were transcribed verbatim using NotebookLM [65]. A thematic analysis was conducted following a multi-stage process [66]. An inductive approach was chosen because in this way, we had the opportunity to record the full range of experiences spontaneously reported by participants. In the first step, the first author independently reviewed all interview transcripts and identified initial themes, which were grouped into categories and subcategories to highlight the key emerging concepts. The main coder’s work was reviewed by the senior researcher in batches of 3–5 interviews, and the coding system was gradually finalized based on feedback and ongoing discussion. The number of disagreements gradually decreased as the coding framework became more refined and the number of interviews increased. As there was only one main coder, no formal agreement (e.g., percent agreement) was calculated.

In some cases, statements could be classified into multiple categories if conceptually required. The codes were reviewed by a senior researcher to ensure consistency, and discrepancies were resolved in consultation with a third member of the research team. We did not use anchor quotes for each code, as it was difficult to identify quotations that reflected the full range of patient experiences. Instead, we used exemplar quotes to illustrate each code without implying complete comprehensiveness. To summarize and organize the collected data, a data matrix was created in Microsoft Excel (Microsoft, Redmond, WA, USA). We counted how many of these impacts identified by the inductive thematic analysis corresponded to items from the EQ-5D-5L, the two bolt-ons, EQ-HWB-9, and the long form of the EQ-HWB. Selected quotes were translated into English to highlight the most relevant insights.

## Results

### Sample characteristics

A total of 21 patients living with EDDs were invited to participate in the study; one withdrew, resulting in a final sample of 20 participants (14 nEDD, 6 sEDD). Most interviews were conducted face-to-face (*n* = 13, 65%). The mean interview duration was 1 h 23 min. No new themes were identified after the sixteenth interview, indicating that data saturation had been achieved. The sample showed a good variability in terms of age, sex, education, clinical characteristics, and current treatments (Table 1).

**Table 1:** Demographic and clinical characteristics of patients

Characteristics	*n* or median	Range or %
Sex assigned at birth		
Female	11	55
Male	9	45
Age	30	20–77
Education		
Primary	1	5
Secondary without certificate	5	25
Secondary with certificate	9	45
College/university	5	25
Employment		
Employed full-time	10	50
Employed part-time	1	5
Unemployed	1	5
Student	5	25
Retired	3	15
Body Mass Index (kg/m^2^)		
Underweight (< 18.5)	1	5
Normal (18.5–24.9)	9	45
Overweight (25.0–29.9)	2	10
Obese (≥ 30)	8	40
Symptoms at birth*		
Erythroderma	14	70
Collodion membrane	11	55
Lamellar scaling	11	55
Ectropion	8	40
Number of body regions affected		
10–15	6	30
≥ 16	14	70
Comorbidities*		
None	7	35
Allergy	5	25
Gastroesophageal reflux disease	3	15
Musculoskeletal disease	3	15
Neurological disease	3	15
Anxiety	2	10
Cancer	2	10
Depression	2	10
Cardiovascular disease	1	5
Crohn’s disease	1	5
Endocrine adrenocortical insufficiency	1	5
Current treatment*		
Topical treatment	20	100
Biological therapy	6	30
Systemic retinoid	3	15
Systemic steroid	1	5
EQ VAS (0-100)	64	35–100
Disease severity (VISI)		
Total core (0–32)	13.74	5–22
Mild severity (0–7)	1	5
Moderate severity (8–15)	8	40
Severe severity (16–23)	11	55
Disease severity (IASI)		
Total score (0–8)	3.67	1–7
Mild severity (0-1.9)	1	5
Moderate severity (2-4.9)	8	40
Severe severity (5-8)	11	55

*Patients may have reported more than one category

IASI, Ichthyosis area severity index; VISI, Visual Ichthyosis Severity Index

### Impacts of epidermal differentiation disorders

In the concept elicitation part, 42 important impacts were identified, which were organized into six main categories: (1) disease course, (2) symptoms and triggers, (3) self-care and treatment-related difficulties, (4) problems with usual activities, (5) mental and social health, (6) other wellbeing aspects (Table 2).

**Table 2:** Important aspects of life affected by epidermal differentiation disorder

Main categories	Subcategories	*n*	%	
Disease course	Genetic origin, onset of disease	20	100	P013: „I’ve had this condition since birth… it’s something I was born with, and I live in this bubble because of it.” (M, 57)
	Hospitalisation, examinations, diagnosis	11	55	P004: „We started going from doctor to doctor, clinic to clinic.” (F, 28)
	Link between skin and other diseases	6	30	P002: „Whenever I got sick, my skin would get inflamed, become more painful, and more erythematous.” (F, 31)
Symptoms and triggers	Discomfort due to inability to sweat* ^▲^	19	95	P002: „I can’t sweat properly, so when I feel hot, I turn red… because I can’t release the heat any other way… where it’s hot, which for me is equivalent to death.” (F, 31)
	Skin scaling, dryness	19	95	P010: „I had very severely flaky skin. And then it came off in sheets… my skin was drier and more cracked” (M, 25)
	Itch* ^▲^	15	75	P005: „When it gets close to bath time, I start to itch around 6 o’clock, so it’s like a mild itch, like my skin is small” (F, 57)
	Erythema	13	65	P005: „ My face gets very red and I can be red almost all day” (F, 57)
	Cold weather	11	55	P020: „In the winter, my skin tends to dry out, and it tends to peel and flake…so when it’s cold, my skin dries out quickly.” (F,25)
	Skin irritation*	10	50	P010: „More irritable… more fluctuating, so more sensitive to changes, such as the weather.” (M, 25)
	Pain* ^┼ ▲^	8	40	P018: „If I don’t pay too much attention to it, the flexure of the skin usually hurts more” (M, 29)
	Water	6	30	P006: „Chlorinated water really messes up my skin — it dries it out completely.” (F, 28)
	Darkening of the skin	5	25	P007: „It will have these very grey scales” (M, 55)
	Exhausted^┼▲^	5	25	P017: „It was exhausting. Everyone else could leave the apartment after 10 minutes if they wanted to, but I needed about an hour to get myself ready, shower, and get dressed. It was time-consuming.” (M, 67)
	Hormonal effects (menstruation, pregnancy, menopause)	5	25	P014: „It was interesting when I was pregnant with my first daughter. My skin was much nicer. I would say it was almost asymptomatic.” (F, 46)
	Diet	4	20	P019: „Well, actually, food is a big contributor, because I’ve noticed that if I eat a lot of carbohydrates, like bread and pasta, I suddenly feel drier, I don’t feel comfortable.” (F, 24)
	Hearing problem ^▲^	4	20	P010: „My hearing started to decline three years ago… and I also noticed changes in my skin – it dries out more quickly, for example, or gets inflamed more easily.” (M, 25)
	Ophthalmological problem (tearing, ectropion)	4	20	P003: „My eyes tear up easily.“(F, 21)
	Stress	2	10	P004: „I’ve been under a lot of stress, and I think that often contributes to the symptoms.” (F, 28)
Self-care and treatment-related difficulties	Previous and current treatments	20	100	P019: „It wasn’t bad, but I felt like the doctor had finally found the cream that worked for me. Since then, my skin has been constantly improving — I receive full-body treatment with the cream, from head to toe.” (F, 24)
	Treatment difficulties, loss of effectiveness	18	90	P005: „When I tried a new body lotion, I could say that there was a noticeable improvement for a month or two, but after a while it didn’t work anymore.” (F, 57)
	Taking a shower, having a bath * ^▲^	16	80	P013: „Showering is not enough because my skin gets inflamed just the same.” (M, 57)
	Time commitment	10	50	P010: „it’s time consuming to apply lotion everywhere after bathing every day…then you have to use lotion after swimming, and then it’s extra time again…in terms of time I’m just running short then I feel that I could have spent this time on something more useful” (M, 25)
	Hair care	7	35	P006: „The doctor recently prescribed a new scalp treatment. It’s working well — my scalp tends to thicken quickly, but with a fine-toothed comb I can now remove the buildup more easily.” (F, 28)
	Side effects of the treatments	6	30	P003: „ I experienced hair loss as a side effect of my treatment.” (F, 21)
Problems with usual activities	Career choice, finding a job, work-related difficulties* ^┼ ▲^	11	55	P017: „I wanted to be a furniture maker, but there’s a lot of dust there, so it wouldn’t have been pleasant, and it influenced my career choice” (M, 67)
	Clothing	10	50	P008: „Clothing is also a bit difficult…Unfortunately, in the summer you have to wear long clothes because it gets ugly down there” (F, 31)
	Holiday and swimming pool activities	10	50	P002: „My skin holds me back in many ways. It affects my quality of life… I can’t go on holiday the way other people can.” (F, 31)
	Cleaning, laundry* ^┼ ▲^	8	40	P005: „When I think about it, it’s a small thing, but I think I do much more laundry than the average person” (F, 57)
	Leisure time activities, sports* ^┼ ▲^	8	40	P006: „I like to do sports, I would like to do sports, but I can’t sweat, so I can’t do them the way I want to.” (F, 28)
	Getting around outside^┼ ▲^	4	40	P001: „At least I feel very bad if I travel, and then I have to scratch here too…because I don’t want anyone to misunderstand.” (F, 77)
	Concentration ^┼▲^	1	5	P017: “It really disturbed me when studying — I had to concentrate hard to keep it from distracting me.” (M, 67)
Mental and social health	Self-acceptance of and adjusting to living with the disease	19	95	P010: „If I focus on my skin condition, I see it as part of me. So just like my other diseases, I try to accept it to the maximum.” (M, 25)
	Stigmatization	16	80	P002: „People don’t come across this condition — most have never seen anything like it. Sometimes I feel like I’m in a circus, the way they stare and point at me.” (F, 31)
	Exclusion or ostracism^▲^	13	65	P007: „Everywhere one goes, people look at one as if one were a leper or as someone who is excluded by society” (M, 55)
	Having to constantly explain my disease to others and that it is not contagious	13	65	P001: „I cannot prove that it is not contagious. They don’t know what it is.” (F, 77)
	Shame	13	65	P001: „I’ll have to be a little shy because I’ll have to cover myself up even in summer.” (F, 77).
	Acceptance by other people ^▲^	7	35	P018:” In high school, at work, I have been fully accepted and had no reservations” (M, 29)
	Romantic relationship problems	6	30	P008: „I don’t have a partner, because of my ichthyosis, in my opinion, I am the only one without family.” (F, 31)
	Support received from other people ^▲^	5	25	P014: „I have two wonderful children, I have a partner, they are a great strength to me” (F, 46)
	Sad/depressed* ^┼ ▲^	2	10	P002: „There have been many times when I felt like it would be easier if I weren’t here.” (F, 31) P004: „This condition broke me down emotionally” (F, 28)
	Frustration ^▲^	5	25	P007: „At the age of nine, I was already starting to understand what was happening, and it caused serious frustration.” (M, 55)
	Self-confidence*	3	15	P015: „The way people treated me as a child — staring, making comments, picking on me — it leaves a mark. It doesn’t exactly help my self-confidence.” (M, 30)
Other wellbeing aspects’	Financial problems	4	20	P014: „Naturally, it’s also a financial burden too, all the creams, and it really matters what you bathe with.” (F, 46)

*HRQoL areas covered by the EQ-5D-5L and the bolt-ons; ^┼^HRQoL or wellbeing areas covered by the EQ-HWB-9; ^▲^HRQoL or wellbeing areas covered by the EQ-HWB long form

Participants spontaneously referred to all EQ-5D-5L and bolt-on dimensions when describing the impact of EDDs, most commonly to discomfort (*n* = 19, 95%), self-care problems (*n* = 16, 80%), and itching (*n* = 15, 75%). Problems with self-confidence were spontaneously mentioned by 15%. Participants spontaneously discussed all EQ-HWB-9 concepts except anxiety and loneliness. Among the 16 items of the EQ-HWB long form that do not appear in the EQ-HWB-9, patients spontaneously mentioned discomfort (*n* = 19, 95%), self-care (*n* = 16, 80%), exclusion (*n* = 13, 65%), acceptance by others (*n* = 7, 35%), supported by others (*n* = 5, 25%), frustration (*n* = 5, 25%), and hearing difficulties (*n* = 4, 20%).

### EQ-5D-5L and bolt-ons

#### Relevance and comprehensiveness, and comprehensibility of the EQ-5D-5L and bolt-ons

Overall, 95% of patients (*n* = 19) found the EQ-5D-5L with bolt-ons comprehensible, useful, and easy to complete: *„It was useful because you could search for yourself in it*,* from a psychological point of view.”*(M, 67). Almost all patients considered the seven dimensions relevant to their condition: *„It covers a wide range of issues related to the disease*,* including psychological and physical aspects*,* as well as symptoms.”*(F, 57). For ‘mobility’, every participant defined it as the ability to walk, climb stairs, and do sports. Over half of the participants (*n* = 11, 55%) interpreted self-care, such as applying treatment, skincare, and choosing the right clothes that do not cause skin irritation. Half of the patients (*n* = 10, 50%) talked about the limitations caused by skin disease when performing usual activities. Pain and discomfort were understood by participants as related dimensions of EDDs, such as skin pain, dryness of the skin, inability to sweat, and sometimes itching. In the case of anxiety and depression, most participants interpreted the dimension in the context of their skin disease. Skin irritation was described as skin inflammation, pain, itching, hives, peeling, sunburn, allergy, sweating, scratching, discomfort, and irritation caused by clothing and laundry detergent. Participants discussed how their condition negatively affected their self-confidence: *„When it comes to self-confidence*,* I’m not doing well. I don’t like stepping out of my comfort zone. It’s probably because of my skin*,* too.”*(F, 28).

#### Order of dimensions

The majority of the patients (*n* = 19, 95%) agreed that the order of the dimensions was acceptable. One patient suggested, *„I would put skin irritation at the top of the list.”*(F, 77).

#### Ranking of dimensions

When asked to prioritize the dimensions in terms of relevance, the majority of patients considered skin irritation (*n* = 15, 75%) as the most relevant, followed by self-confidence and pain/discomfort (*n* = 10, 50% each). Mobility was most frequently mentioned as the least relevant dimension (*n* = 10, 50%). Interestingly, skin irritation was considered by only one participant, while pain/discomfort was not identified as at least relevant by any of the patients (Table 3).

**Table 3:** Most and least relevant dimensions in the EQ-5D-5L and bolt-ons, and EQ-HWB-9

EQ-5D-5L + bolt-ons					EQ-HWB-9				
	Most relevant		Least relevant			Most relevant		Least relevant	
Item	n	%	n	%	Item	n	%	n	%
Mobility	2	10	10	50	Day-to-day activities	14	70	2	10
Self-care	8	40	1	5	Getting around inside or outside	7	35	7	35
Usual activities	7	35	3	15	Exhaustion	1	5	5	25
Pain/discomfort	10	50	0	0	Loneliness	5	25	3	15
Anxiety/ depression	3	15	5	25	Concentrating/thinking clearly	1	5	4	20
Skin irritation	15	75	1	5	Anxiety	0	0	1	5
Self-confidence	10	50	5	25	Sadness/depression	6	30	0	0
					Control	0	0	6	30
					Pain	12	60	2	10

Each patient could have selected more than one item as the most or least relevant dimension

#### Conceptual overlaps between the dimensions

None of the 20 participants identified overlapping content between the dimensions, except one patient who noted a potential association between pain and skin irritation: *„Obviously*,* if your skin hurts*,* it also irritates*,* so there is some overlap. But in my opinion*,* both are important.”*(M, 20).

**Table 4:** Suggested changes to the EQ-5D-5L and bolt-ons

Themes	*n*	%	Example quote
**Mobility**			
Add more examples to the dimension	2	10	P012: „In order to make it more understandable if someone is filling it in for themselves, it would be more useful to use examples; sport, everyday walking, running, athletic activity” (M, 20)
Specify the meaning of mobility	2	10	P010: „I would like to clarify whether we are talking about simple everyday walking, or doing sport, because sport is part of someone’s everyday life” (M, 25)
**Self-care**			
Add more examples to the dimension	2	10	P010: „In brackets I would add different activities, direct; for example, if I use different body lotions, indirect; clothing” (M, 25)
Omission of dressing	1	5	P018: „I wouldn’t actually put it in there, because the fact is that this disease doesn’t affect dressing” (M, 29)
Split into 2 questions: washing self and dressing self	1	5	P008: „I would ask about washing self and dressing self separately” (F, 31)
**Usual activities**			
Add more examples to the dimension	1	5	P012: „In order to make it more understandable if someone is filling it in for themselves, it would be more useful to use examples: hobby, writing, typing” (M, 20)
Split into questions: work, study, housework, family, or leisure activities	1	5	P012: „It could be broken down into several questions, because, the usual activities include work, study, homework, and leisure activities and here, for instance, you could ask separately about work, even leisure activities in my opinion, it would be better to think about this” (M, 20)
**Pain/discomfort**			
Split into 2 questions	6	30	P006: „It would be worth asking separately, because the truth is that I can feel discomfort without pain” (F, 28)
Add more examples to the dimension	2	10	P010: „I would make it a bit more concrete, like the usual activities, so I would add different activities in brackets such as in the case of pain, on the one hand something that I cannot influence, e.g. the weather, and on the other hand something that can be influenced, e.g. dressing” (M, 25)
Merge discomfort with anxiety/ depression	2	10	P015: „Well, maybe discomfort, anxiety, depression – those are similar terms, perhaps. And then anxiety, depression, discomfort – those are one question, and then pain is a separate question. (M, 30)
Omission of pain	1	5	P014: „I would specifically eliminate the pain… ” (F, 46)
Rephrase the word discomfort as ‘unpleasant symptoms’	1	5	P003: „I would change the word “discomfort” for “unpleasant symptoms”, because I don’t usually feel discomfort, but rather inconvenience” (M, 21)
**Anxiety/depression**			
Split into 2 questions	2	10	P012: „Anxiety/depression, less problematic, but I think it would be better to ask separately” (M, 20)
Add a simpler word next to depression	1	5	P014: „I don’t know how people understand depression in this way in today’s world. .Well, maybe they could write something behind it, I just don’t know what the translated version of this medical word would be” (F, 46)
Examples, longer descriptions	1	5	P010: „I would expand the question, anxiety: when you are very upset about something, you think a lot, you worry a lot. Depression: you feel very bad in the long term, you have negative thoughts, you have very little motivation, you feel hopeless about your future, you are in a bad mood, you don’t feel like doing anything” (M, 25)
**Skin irritation**			
Add more examples to the dimension	1	5	P010: „To use the example of skin irritation, tingling, skin pain, as an example of the usual activity question” (M, 25)
Move up skin irritation to be the first item in the EQ-5D-5L	1	5	P001: „I would put skin irritation at the top of the list” (F, 77)
**Self-confidence**			
Add more examples to the dimension	1	5	P012: „As an example, sure you are of yourself when making decisions in front of other people or about other people, how insecure you are, how self-conscious you are, that I will not be discredited or thought badly of” (M, 20)

#### Suggested changes

Taken together, 14 participants (70%) suggested changes to one or more of the seven dimensions (Table 4). There were two suggestions related to the bolt-ons, including moving skin irritation to be the first dimension and adding more examples beyond itching, such as tingling.

#### Missing concepts

In total, 8 missing concepts were identified by 12 participants (60%). These were classified into two main groups: (1) EDD-related concepts, and (2) other aspects (Online Resource 1). The most frequently reported missing concepts were difficulties related to treatment. Other aspects, such as romantic relationships, were raised by only one patient each.

#### EQ VAS

When interpreting the EQ VAS, seven participants (35%) focused specifically on their skin symptoms such as pain, dryness, and scaling and viewed ‘the best health’ as being asymptomatic: *“The best health condition would be a miraculous achievement*,* such as if my skin were to heal or if the treatment produced a dramatic improvement.“*(F, 28). Nine participants (45%) interpreted the two endpoints in general, such as severe illness or near death (='0'). Four patients (20%) considered both their skin and general health when responding.

### EQ-HWB-9

#### Relevance and comprehensiveness, and comprehensibility of the EQ-HWB-9

Overall, 14 patients (70%) found the questionnaire appropriate for expressing their experiences related to EDDs: *„It completely covers the problems of this disease in my everyday life”.* (M, 29). However, four patients noted it is less related to EDDs: *„I didn’t think about my illness at all; I was focused on my physical and emotional state.“*(F, 57). Fifteen patients reported that the EQ-HWB-9 was clear and easy to understand. The ‘day-to-day activities’ item was understood as intended (e.g., housework, work, leisure time activities) by 18 participants (90%). One participant found the ‘getting around inside or outside’ item difficult to interpret because of the examples in parentheses; *„I didn’t understand this at first because I don’t use such tools.”*(F, 31). Some patients (n = 5, 25%) interpreted this item as referring not only to physical movement but also to the use of public transport. Most patients (n = 13, 65%) associated ‘exhaustion’ primarily with work-related fatigue, while ’loneliness’ was generally linked to being alone. For the anxiety item, patients often referred to their skin condition as a source of anxiety. Many patients (n = 16, 80%) interpreted the ‘sad/depressed’ item in terms of deeper emotional challenges such as the loss of a loved one, family issues, or low mood. ’Control’ was interpreted by patients in the following way: „*I am dependent on someone else to tell me what I can do.”*(M, 30), without mentioning their skin condition. Two patients had difficulty distinguishing between the response options ‘a little of the time’ and ’sometimes’. Interpretation of the physical pain item varied widely, including skin-related symptoms (e.g. itching and burning), but also other pains such as headache and back pain. Three patients expressed difficulties in responding to the item on physical pain: *„The physical pain question*,* it seems like you have to answer 5 different questions*,* it was confusing.”*(F, 77).

#### Order of dimensions

There was general agreement among 15 patients that the order of the items was appropriate. Two patients suggested moving the physical pain item to the beginning of the questionnaire, and one participant commented the following: *„The fact that it was difficult to concentrate or think clearly*,* I would probably put that last because my skin condition does not affect that.”*(F, 21).

#### Ranking of dimensions

The majority of patients considered ‘day-to-day activities’ to be the most relevant item (n = 14, 70%), followed by ‘physical pain’ (n = 12, 60%). None of the participants mentioned ‘anxiety’ or ‘control’ as the most relevant items. ‘Getting around inside or outside’ was rated as the least relevant item (n = 7, 35%), closely followed by control (n = 6, 30%) (Table 3).

#### Suggested changes

In the EQ-HWB-9, 13 participants suggested changes, with modifications proposed for all but three items (day-to-day activities, getting around inside or outside, and exhaustion) (Table 5). The use of simpler colloquial language was recommended by one participant. Three participants recommended splitting ‘sadness/depression’ into two separate items. Three participants suggested combining loneliness with other items (sadness/depression, concentrating/thinking clearly, anxiety). When patients were asked about recent wording changes in EQ-HWB-9, 80% (n = 16) preferred ’only occasionally’ (experimental) over ’a little of the time’ (experimental modified), and 70% (*n* = 14) favored ’moderate difficulty’ (experimental modified) over ’some difficulty’ (experimental).

**Table 5:** Suggested changes to the EQ-HWB-9

Themes	*n*	%	Example quote
**Loneliness**			
Combine loneliness and sad/depressed into one item	2	10	P015: „Loneliness, sad, depressed, they’re probably somewhat similar to each other, so they’re not the same, of course, but you could ask the three together” (M, 30)
Combine loneliness, concentration, worried, anxious into one item: “how one overall feels”	1	5	P005: „Then let’s look at loneliness, concentration, worried, and anxious, these could be asked in one question, I think, how did you feel or what was your state of mind?” (F, 57)
**Concentrating/thinking clearly**			
Move up, concentrating/thinking clearly, to be the last item in the questionnaire	1	5	P003: „The fact that it was difficult to concentrate or think clearly, I would probably put that last because my skin condition does not affect that.” (F, 21)
**Anxious**			
Omission of worried (Hungarian specific)∗	2	10	P013: „Worried is therefore like anxious, because worried is upset by something, so it is also anxiety. So they’re the same thing, so ask about anxious” (M, 57)
Omission of anxious	1	5	P010: „I prefer to think of anxious as worried. So to me, the two are basically synonyms” (M, 25)
**Sadness or depression**			
Split into 2 questions	3	15	P007: „Depression is not typical for me, so I would like to split into 2 questions” (M, 55)
Omission of sad	1	5	P013: „The two concepts are almost the same, so if someone is sad, they will fall into themselves, so they will be depressed. I would ask only about the depression” (M, 57)
Replace depression with melancholy	1	5	P015: „I think melancholy might be a more correct term” (M, 30)
Use bipolar scale instead with motivation and depression as endpoints	1	5	P010: „I would draw such a scale. Maybe it starts with a lack of motivation, and then gradually you get sad, and then later on you get depressed” (M 25)
**Control**			
Delete the dimension	1	5	P018: „The question of having no influence on your daily life and the question of daily activity are in some way intertwined; they are opposites, it would be enough to ask about daily activity” (M, 29)
**Pain**			
Move up pain to be the first item in the questionnaire	2	10	P007: „Perhaps you would put the questions about the mental state at the end, and the question about the physical pain caused in the last seven days, so I would put that one at the front”(M, 55)
Rephrase physical pain as bodily pain	1	5	P014: „Maybe physical pain can be replaced by bodily pain” (F, 46)
**Others**			
Using simpler, colloquial language	1	5	P001: „It should be phrased kind of in plain kitchen talk, a bit like how a country folk would say it” (F, 77)
Use complete sentences for question formulation instead of stems	1	5	P012: „This seems a bit strange, that in the past week, there was an occurrence, and then the sentence ends with a colon… like the sentences are kind of cut off, so to speak. I think maybe the previous one [EQ-5D-5L + bolt-ons] is a bit better because, in that case, everything is written out separately, and it’s clearer that way.” (M, 20)

*The Hungarian translation of the anxiety EQ-HWB-9 item uses both the terms ‘worried’ and ‘anxious’

#### Missing concepts

Altogether, 7 missing concepts were mentioned by 12 patients (Online Resource 2). Similar to the EQ-5D-5L and bolt-ons, the difficulties of treatment were the most commonly identified missing concept (*n* = 5, 25%), followed by skin appearance (*n* = 4, 20%). One patient mentioned social relationships, and another mentioned self-confidence as missing.

#### EQ-HWB VAS

**Fig. 1:**
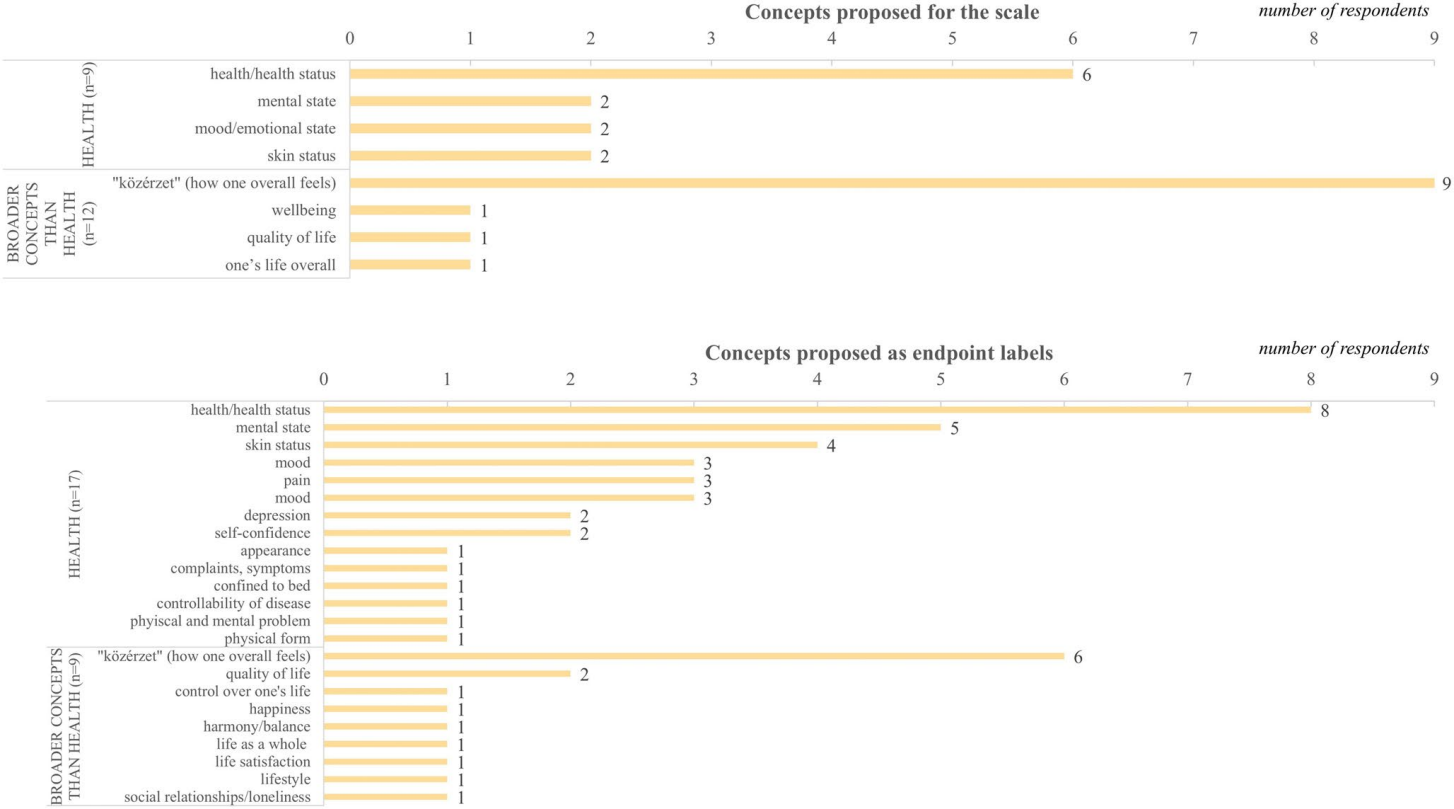
Proposed concepts for the ’blank’ EQ-HWB VAS and its endpoints

Participants mentioned seven different concepts regarding what a potential EQ-HWB VAS should aim to measure (Fig. 1). Nine patients referred to health-related aspects, and 12 mentioned broader concepts than health. The most frequently suggested terms were how one overall feels (in Hungarian: ‘közérzet’) (*n* = 9, 45%) and health/health status (*n* = 6, 30%).

When asked to specify the EQ-HWB VAS endpoints, altogether, 32 distinct concepts were identified. Seventeen (85%) patients mentioned health-related concepts, with health/health status (*n* = 8, 40%), mental state (*n* = 5, 25%), and skin status (*n* = 4, 80%) being the most common. Nine (45%) patients suggested broader concepts, where how one overall feels (*n* = 6, 30%) was most frequently cited, followed by quality of life (*n* = 2, 10%).

### Recall period and comparison of the two instruments

No patients spontaneously reported any issues with the recall period. However, asking about the optimal recall period to measure EDD impacts, one patient suggested 3 days, and three patients considered one week to be suitable. Most patients proposed a longer recall period due to the fluctuation of their symptoms. More than half of the participants (*n* = 11, 55%) suggested one month. Three patients preferred a much longer recall period of half a year or even one year.

When comparing the EQ-5D-5L with two bolt-ons and EQ-HWB-9, a total of 12 patients preferred the item-by-item response format, 6 preferred the grid, and 2 had no preference. Regarding response options, the majority of patients considered the severity scale (*n* = 16, 80%) more appropriate than the frequency for expressing EDD-related issues. Overall, 16 patients found the EQ-5D-5L with bolt-ons more useful to express the impacts of EDDs, while 4 preferred the EQ-HWB-9 (e.g., *“it [EQ-5D-5L + bolt-ons] is clearly better in my opinion because it asks much better questions about problems related to the disease.”*(F, 25).

## Discussion

Participants considered the EQ-5D-5L with bolt-ons and EQ-HWB-9 to be relevant and appropriate for measuring the impact of EDDs, traditionally known as ichthyoses, on their lives. Before completing the questionnaires, the patients spontaneously mentioned several health and wellbeing dimensions covered by EQ-5D-5L, bolt-ons, and EQ-HWB-9 as affected by EDDs (such as itching, discomfort, and day-to-day activities). In the EQ-5D-5L, ‘skin irritation’ was identified as the most relevant dimension, while in the EQ-HWB-9, most participants highlighted ‘day-to-day activities’ as the most important item in EDDs. The conceptually related items in the EQ-5D-5L and EQ-HWB-9 were not always interpreted in the same way by participants. For instance, ‘pain’ in the EQ-5D-5L was more often associated with skin-related symptoms, but in EQ-HWB-9, it was usually understood as pain affecting any organ or body area, not only the skin. This reflects the differing focus of these instruments: the EQ-5D-5L asks about health problems, while the EQ-HWB-9 seems to encourage respondents to think about general health and wellbeing more broadly.

Patients found the two EQ-5D-5L bolt-ons relevant and appropriate for expressing impacts related to EDD, as was also the case in other dermatological conditions in previous studies [13, 17, 19]. Similar to the results for the populations living with psoriasis, atopic dermatitis, and chronic urticaria, ‘skin irritation’ was the most relevant dimension among patients with EDD [13, 16, 17]. While some of these previous studies in other skin conditions found potential partial conceptual overlap between pain/discomfort and skin irritation, this was mentioned by only one patient in our study, and no one considered self-confidence and anxiety overlapping. This may be explained by the specific symptom profile of EDDs, where pain (due to fissures) and discomfort (temperature sensitivity) often occur as separate and qualitatively different sensations from itching.

Although the experimental modified version of the EQ-HWB-9 was generally found clear, relevant, and comprehensive by most patients with EDDs, our study identified several smaller points for further improvement of the descriptive system. Understanding problems were found with the ’getting around inside or outside’ and ’pain’ items. The comprehensibility problems with the examples for using aids for the mobility item align with findings from earlier content validity studies with carers in other countries [24, 28]. In these earlier studies, participants often focused on examples involving mobility aids (e.g. wheelchair) and misunderstood the question, interpreting it as asking whether they used such devices, or were unsure how to respond if they did not. In our study, EDD patients sometimes misunderstood this item and considered it as a reference to problems using public transportation, focusing on others seeing them scratching rather than on actual physical mobility difficulties.

Participants welcomed the addition of a potential EQ-HWB VAS to complement the EQ-HWB-9, noting that it would help them to summarize how they had felt over the past seven days. The large number of endpoint labels and concepts proposed for the EQ-HWB VAS suggests that it should capture broader aspects beyond health, but this also indicates that development and decision-making will be complex. Future research is needed to validate these findings in other languages and target populations (e.g., caregivers and social care users), define the optimal concepts and endpoints for a potential VAS for the EQ-HWB, and to qualitatively and quantitatively test alternative versions.

Earlier studies and our current findings indicate that the EQ-5D-5L with the two bolt-ons from the experimental EQ-5D Bolt-on Toolbox is largely comprehensive in dermatological conditions, including EDD [15–21]. While additional constructs could enhance content validity for dermatological conditions, most are either condition-specific (e.g. treatment difficulties in EDD) or not HRQoL dimensions (e.g. financial burden of the disease), therefore would not fit the measurement framework of the EQ-5D-5L and could reduce comparability across diseases. For the EQ-HWB-9, most missing concepts did not align with the instrument’s broader health and wellbeing focus (e.g. treatment difficulties in EDD, skin appearance). One patient mentioned social relationships as a missing concept. In a previous study, although in a completely different context, Australian carers of aged care recipients suggested potential missing EQ-HWB long-form items, including social interaction, similar to our findings [24]. However, the loneliness item is expected to capture the social domain according to the development framework, and because it was raised by only few participants, this does not appear to justify any actionable changes at this time.

There are several limitations to this study. First, all of the patients were recruited from a single dermatology clinic, which may limit the generalizability of the findings. Second, the order of the questionnaires was not randomized: the EQ-5D-5L bolt-ons was always present before the EQ-HWB-9 questionnaire, which may have influenced the responses to the EQ-HWB-9. Third, no triangulation (e.g. multiple researchers coding the same interviews or obtaining perspectives on the instrument from another group, such as carers) was performed. Finally, the Hungarian version of the EQ-HWB-9 used in this study was not the final version; it was the second consensus version, which underwent two forward and two backward translations and review by the EuroQol’s Version Management Committee, but not cognitive debriefing. Importantly, the translations of the response levels ’a little of the time’ (previously ’*ritkán’* to ’*elvétve’*) and ’sometimes’ (previously ’*néha’* to ’*néhányszor’*) were changed after cognitive debriefing. The same applies to the text in parentheses after the ’getting around inside or outside’ item. Therefore, the problems reported for these items may not apply to the final version. The Hungarian translation of ‘getting around’ as *‘közlekedni*’ may also need to be reconsidered to avoid associations with public transportation.

Overall, both instruments appear to be relevant to this population; however, they serve different purposes. Patients’ feedback confirmed that to measure direct disease impact and health aspects, the EQ-5D-5L with bolt-ons seems more suitable, while the EQ-HWB-9 measures impact on overall life, including broader aspects beyond health. For estimating quality-adjusted life years in cost-utility analyses, the bolt-ons may enhance the sensitivity of the EQ-5D-5L by capturing symptom-specific impacts.

## Conclusions

This is the first study to assess the content validity of two bolt-ons from the experimental EQ-5D Bolt-on Toolbox and the experimental EQ-HWB-9 in the EDD population. Scientific evidence from this study contributes to the refinement and finalization of these instruments. Furthermore, this study provides the first exploratory qualitative results about potential endpoints for an EQ-HWB VAS.

## Supplementary Information

Below is the link to the electronic supplementary material.

Supplementary Material 1

## Data Availability

The data that support the results of this study are available upon reasonable request from the corresponding author.
