# Supplementary material for: Qualitative testing of the EQ-HWB-9 with a ‘blank’ EQ-HWB VAS and EQ-5D-5L with skin bolt-ons in a rare genetic skin disease population
Source: Qual Life Res. 2026 Feb 12;35(3):69. doi: 10.1007/s11136-026-04177-0 (PMC12901120; doi:10.1007/s11136-026-04177-0)
Supplement: Supplementary file 1 — Supplementary Material 1 [file 11136_2026_4177_MOESM1_ESM.docx]

**Supplementary Material**

**Quality of Life Research**

**Qualitative testing of the EQ-HWB-9 with a ’blank’ VAS and EQ-5D-5L with skin bolt-ons in a rare genetic skin disease population**

*Contents*

[**Online Resource 1 Missing concepts from the EQ-5D-5L and bolt-ons** 2](#_Toc205915711)

[**Online Resource 2 Missing concepts from the EQ-HWB-9** 3](#_Toc205915712)

# **Online Resource 1 Missing concepts from the EQ-5D-5L and bolt-ons**

| **Type of concepts** | **Concepts** | **Example quote** | **Patients mentioning each concept** | | **Patients mentioning ’type of concept’** | |
| --- | --- | --- | --- | --- | --- | --- |
|  |  |  | n | % | n | % |
| Epidermal differentiation disorder related concepts | Difficulties of the treatment | P020: „A patient with ichthyosis should definitely include how they treat their skin” (F, 25) | 2 | 10 | 4 | 20 |
|  | Doctor-patient relationship | P005: „I'm thinking about what is the doctor-patient relationship, how much does the health care system care about the burden of disease?” (F, 57) | 1 | 5 |  |  |
|  | The effect of water on the skin | P009: „Water is also very drying, so let's say it's something like this, the effect of water on the skin” (F, 21) | 1 | 5 |  |  |
|  | The effect of weather on the skin | P009: „The effect of the weather on your skin is that I have a very difficult time in the sun, which I think is partly due to ichthyosis”( F, 21) | 1 | 5 |  |  |
| Other aspects | Diet | P007: „Nutrition, I myself have read a lot about this and we get relatively little carotene with vitamin A in our diets”(M, 55) | 1 | 5 | 4 | 20 |
|  | Financial burden of disease | P014: „How much of a financial burden for the family?” (F, 46) | 1 | 5 |  |  |
|  | Impact on studying and fitting in at the workplace | P013: „Learning or fitting in at the workplace” (M, 57) | 1 | 5 |  |  |
|  | Romantic relationships | P001: „Man is a social being. So I'm thinking about relationships because this disease has external signs, I want someone to fall in love with me regardless of the fact that they touch me and I may not have the same skin touch, so how does the disease affect relationship?” (F, 77) | 1 | 5 |  |  |

# **Online Resource 2 Missing concepts from the EQ-HWB-9**

| **Type of concepts** | **Concepts** | **Example quote** | **Patients mentioning each concept** | | **Patients mentioning ’type of concepts’** | |
| --- | --- | --- | --- | --- | --- | --- |
|  |  |  | n | % | n | % |
| Epidermal differentiation disorder related concepts | Difficulties of the treatment | P001: „So, for instance, how can I treat the ichthyosis, the creaming? For example, how can I put it on my back, or do I have any kind of a remedy?”(F, 77) | 5 | 25 | 10 | 50 |
|  | Skin appearance | P003: „When we talk about ichthyosis, the skin appearance over the last seven days, say, what it looked like or whether it caused any difficulties”(F, 21) | 4 | 20 |  |  |
|  | Onset of new symptoms (e.g. dry skin, irritation) | P009: „Dryness or rash, whether it was new symptoms. Because I sometimes notice, that I have a new patch, I don't even know where it came from or when it came from”(F, 21) | 3 | 15 |  |  |
|  | Impact of treatment on daily activities | P002: „Getting treatment is how much it affects everyday life...I have a hard time washing clothes because of it” (F, 31) | 1 | 5 |  |  |
| Other aspects | Self-confidence | P012: „I'm missing, for example, what was such a good question in the previous questionnaire, is this question about self-confidence” (M, 20) | 1 | 5 | 2 | 10 |
|  | Social relationships | P002: „The treatment is how much it affects either your daily life or your relationships.....the body cream that I use it has very, very greasy effect and I don't like it when other people touch me when it hasn't been absorbed yet....and I don't like to kiss a family member or my partner when I'm still covered in a thick layer of cream that sticks” (F, 31) | 1 | 5 |  |  |
|  | Solitary or social personality type | P014: „ I would phrase the question as whether they feel more like a solitary or a social type. So, in the past week, have they felt more lonely, or have they been in the company of others more often? (F, 46) | 1 | 5 |  |  |
